# Supplementary material for: Epidemiology of Δ8THC-Related Carcinogenesis in USA: A Panel Regression and Causal Inferential Study
Source: Int J Environ Res Public Health. 2022 Jun 23;19(13):7726. doi: 10.3390/ijerph19137726 (PMC9265369; doi:10.3390/ijerph19137726)
Supplement: Supplementary file 1 [file ijerph-19-07726-s001.zip › Supplementary FIgures captions.pdf]

Supplementary Figure Captions

Figure S1. Temporal trends in selected cannabinoid concentrations in USA FDA seizures.

Figure S2. Map-graph of estimates of Δ8THC concentrations across USA over time. (Estimates derived as described in Methods section).

Figure S3. Pannelled scatterplots of age-standardized rates of selected cancers as a function of tobacco exposure.

Figure S4. Pannelled scatterplots of age-standardized rates of selected cancers as a function of alcohol use disorder incidence.

Figure S5. Pannelled scatterplots of age-standardized rates of selected cancers as a function of Δ9THC exposure.

Figure S6. Time-aggregated rates of selected cancers in highest and lowest tobacco exposure quintiles.

Figure S7. Time-aggregated rates of selected cancers in highest and lowest alcohol use disorder incidence quintuiles.

Figure S8. Time-aggregated rates of selected cancers in highest and lowest Δ9THC exposure quintiles.

Figure S9. Time-aggregated rates of selected cancers in highest and lowest Δ8THC exposure quintiles.

Figure S10. Graphical summary of inverse probability weighted additive panel models by covariate. (A) numbers of cancers identified, (B) total negative P-value of exponents and (C) total minimum E-values.

Figure S11. Graphical summary of inverse probability weighted interactive panel models at two years lag by covariate. (A) numbers of cancers identified, (B) total negative P-value of exponents and (C) total minimum E-values.

Figure S12. Heatmap of substance martginal effects in additive panel model by tumour type.

Figure S13. Heatmap of substance martginal effects in interactive panel model at two years of lag by tumour type.

Figure S14. Heatmap of substance martginal effects in interactive panel model at four years of lag by tumour type.
