# Supplementary material for: Epidemiology of Δ8THC-Related Carcinogenesis in USA: A Panel Regression and Causal Inferential Study
Source: Int J Environ Res Public Health. 2022 Jun 23;19(13):7726. doi: 10.3390/ijerph19137726 (PMC9265369; doi:10.3390/ijerph19137726)
Supplement: Supplementary file 1 [file ijerph-19-07726-s001.zip › Supplementary Figures S1-S14_2.pdf]

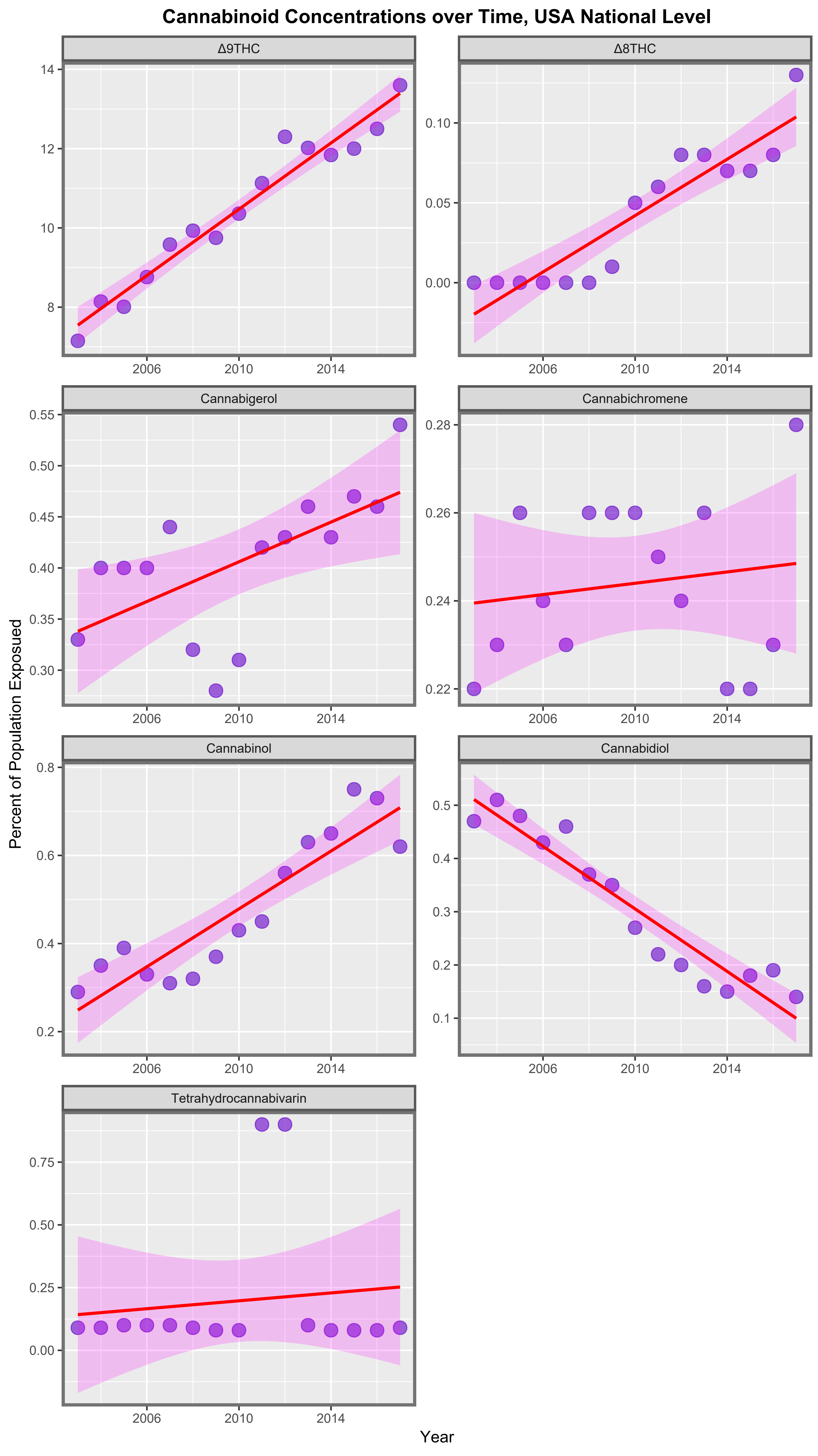
Cannabinoid Concentrations over Time, USA National Level
Δ9THC
Δ8THC
Cannabigerol
Cannabichromene
Cannabinol
Cannabidiol
Tetrahydrocannabivarin
Percent of Population Exposued
Year

..8THC Exposure Across USA by State Over Time

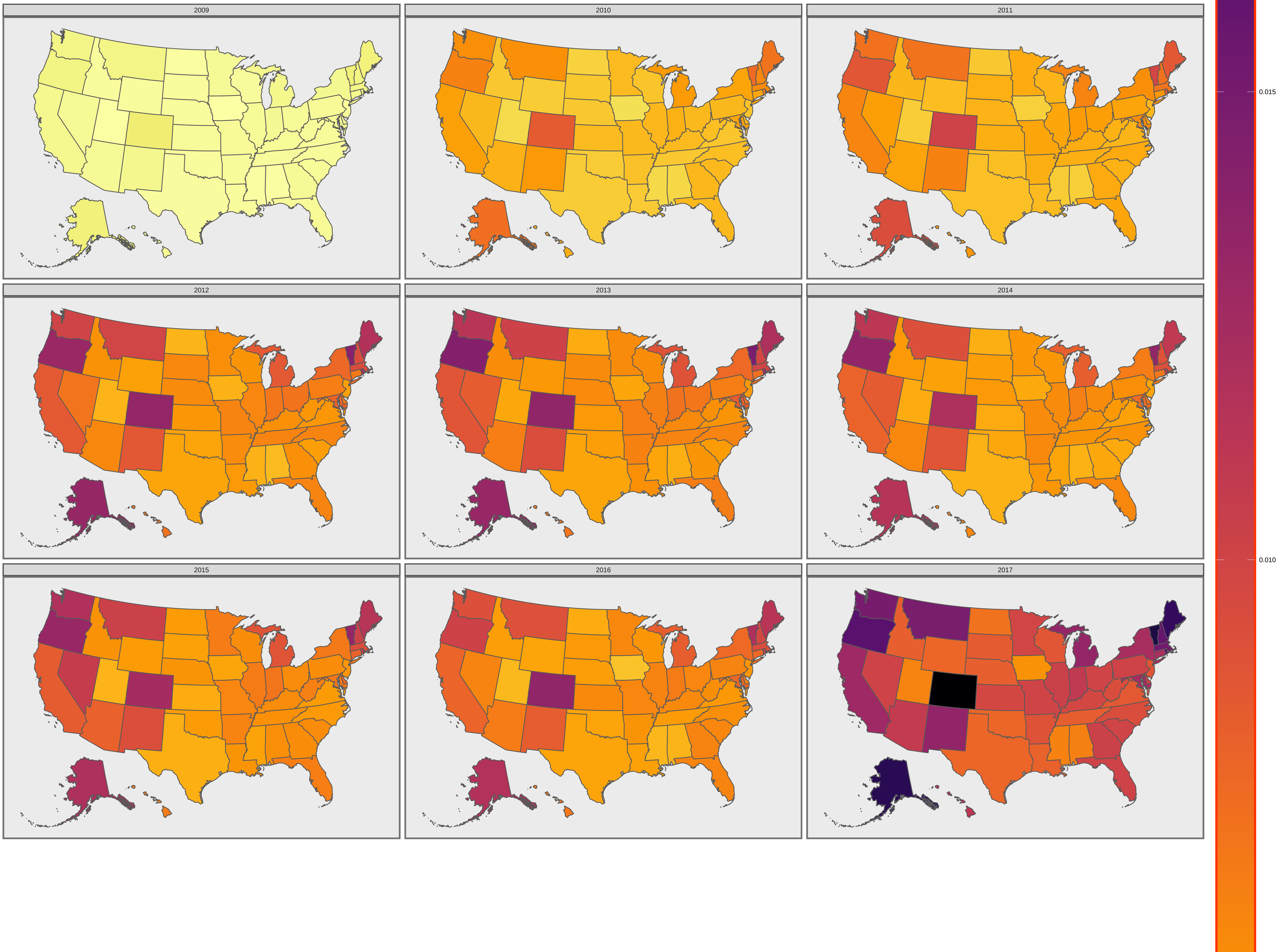

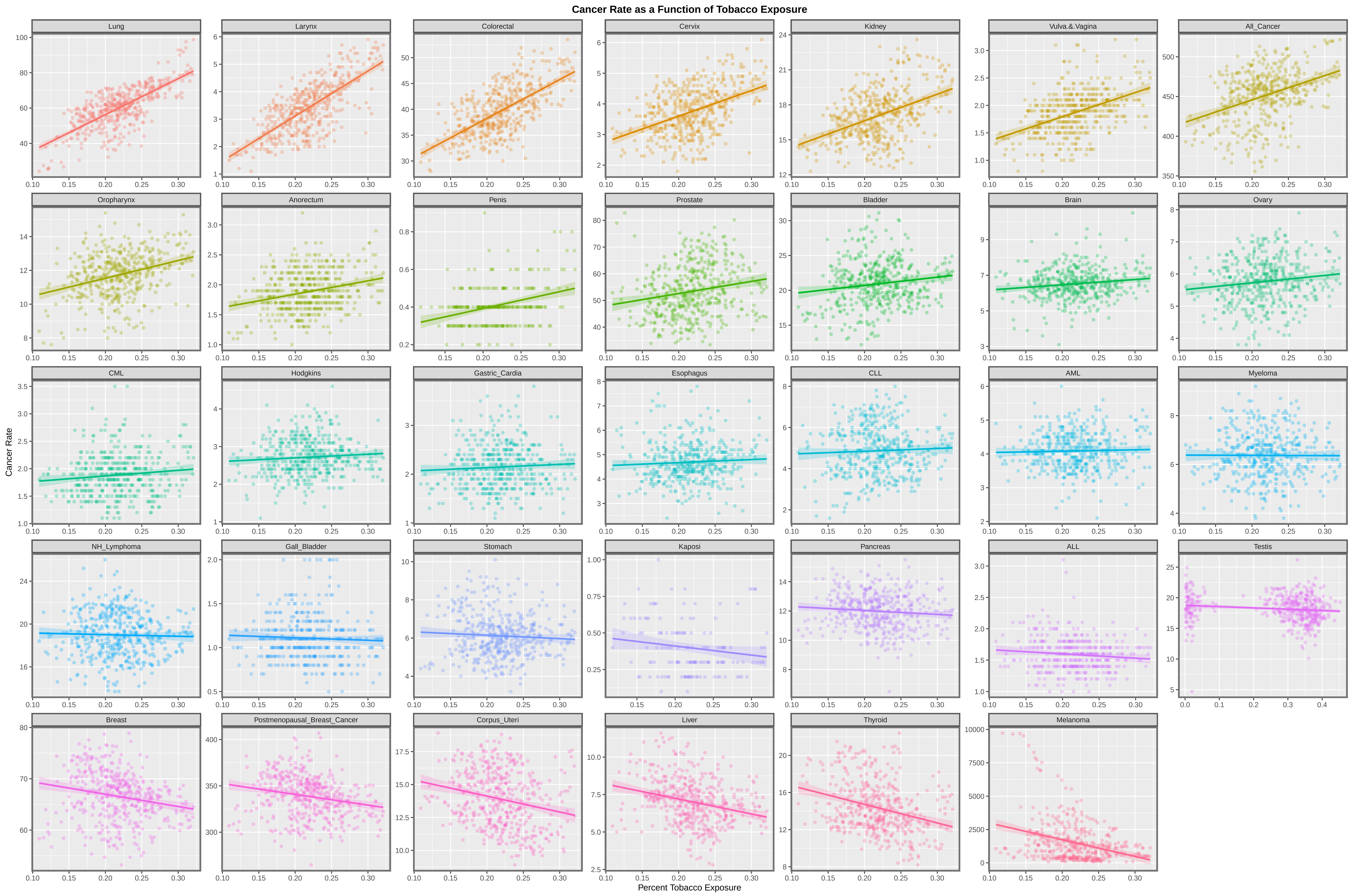
Cancer Rate as a Function of Tobacco Exposure
Lung
Larynx
Colorectal
Cervix
Kidney
Vulva.&.Vagina
All_Cancer
Oropharynx
Anorectum
Penis
Prostate
Bladder
Brain
Ovary
CML
Hodgkins
Gastric_Cardia
Esophagus
CLL
AML
Myeloma
NH_Lymphoma
Gall_Bladder
Stomach
Kaposi
Pancreas
ALL
Testis
Breast
Postmenopausal_Breast_Cancer
Corpus_Uteri
Liver
Thyroid
Melanoma
Cancer Rate
Percent Tobacco Exposure

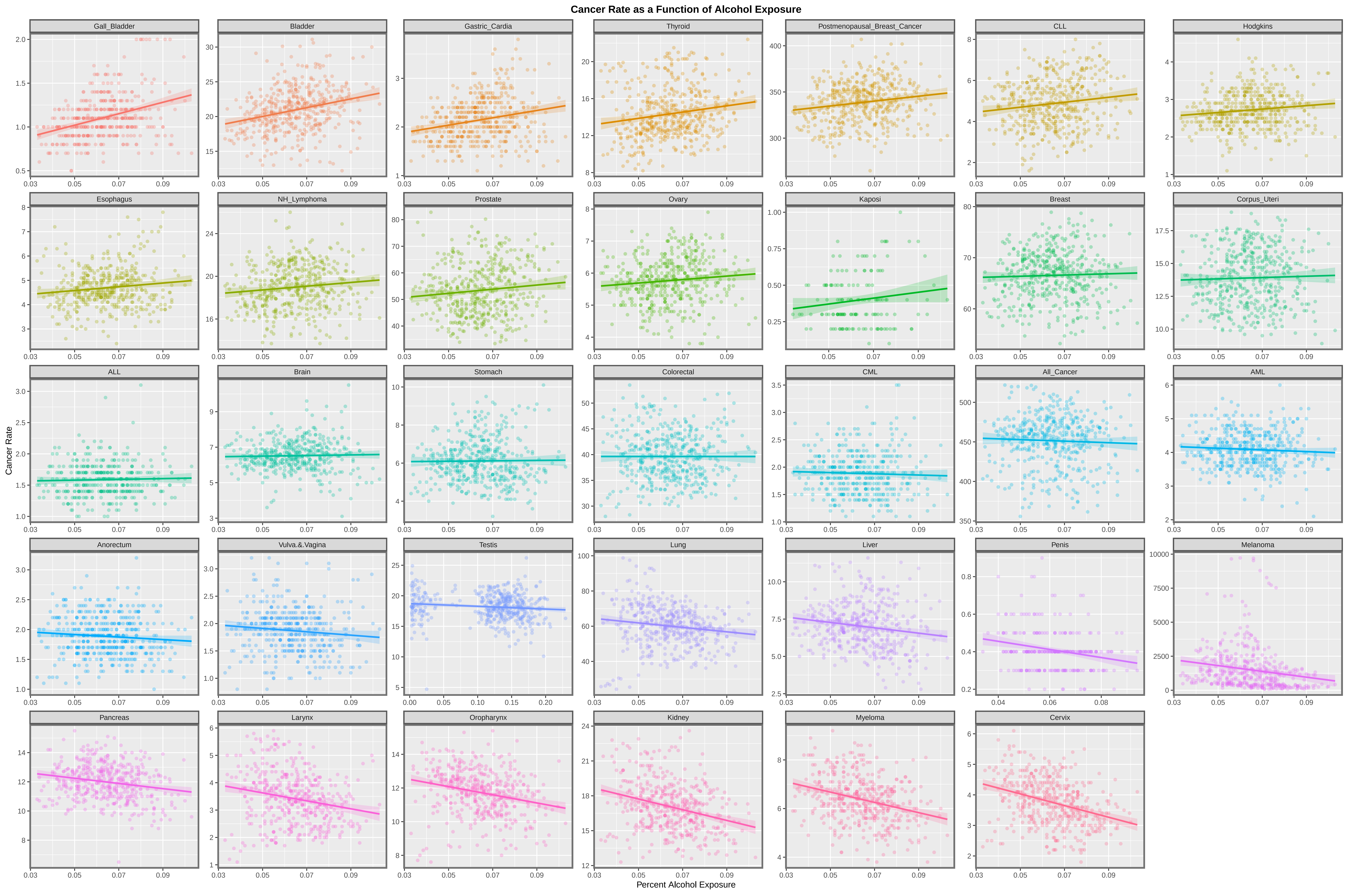
Cancer Rate as a Function of Alcohol Exposure
Gall_Bladder
Bladder
Gastric_Cardia
Thyroid
Postmenopausal_Breast_Cancer
CLL
Hodgkins
Esophagus
NH_Lymphoma
Prostate
Ovary
Kaposi
Breast
Corpus_Uteri
ALL
Brain
Stomach
Colorectal
CML
All_Cancer
AML
Anorectum
Vulva.&.Vagina
Testis
Lung
Liver
Penis
Melanoma
Pancreas
Larynx
Oropharynx
Kidney
Myeloma
Cervix
Cancer Rate
Percent Alcohol Exposure

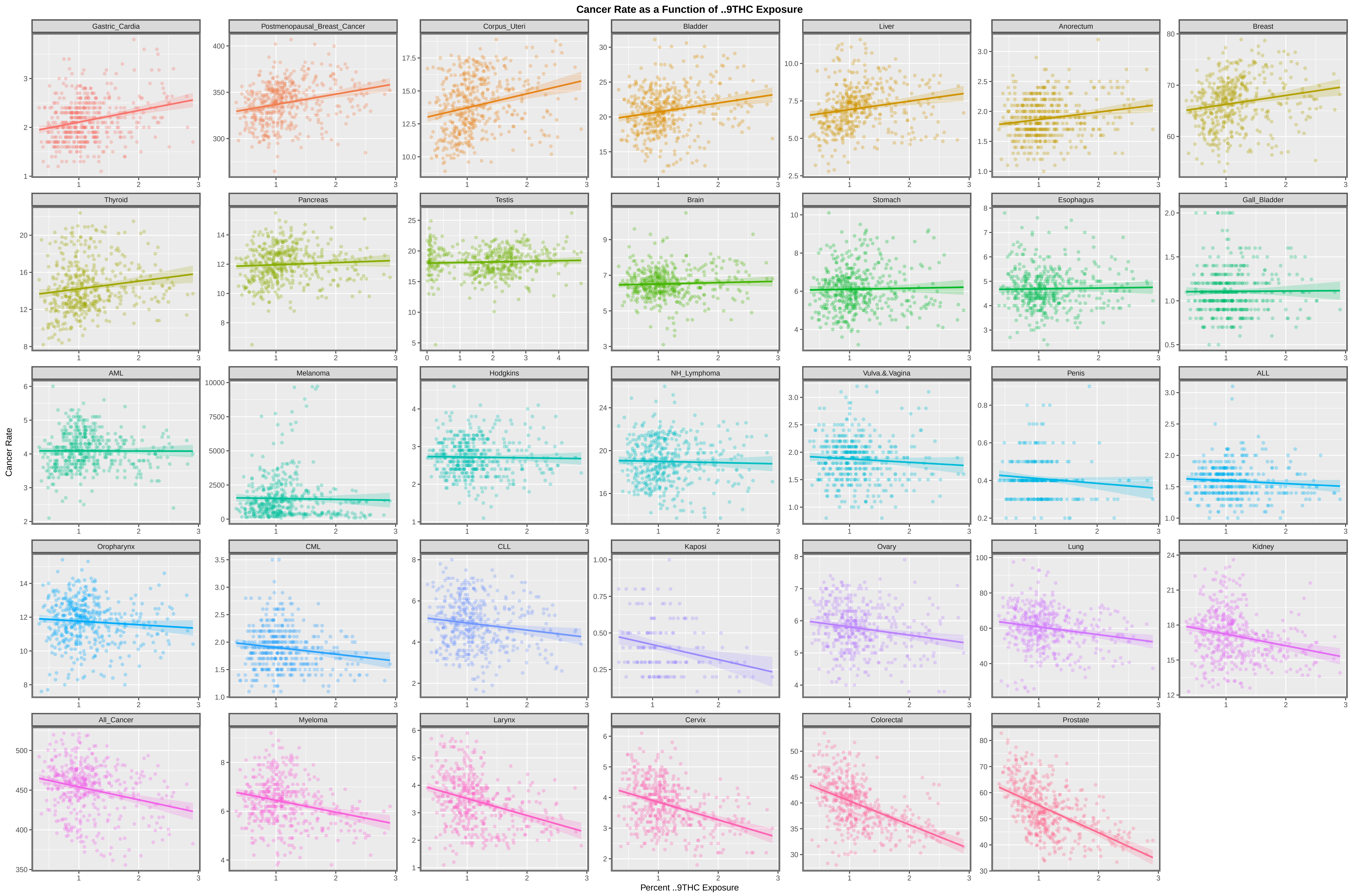

Cancer Rate as a Function of ..9THC Exposure
Gastric_Cardia
Postmenopausal_Breast_Cancer
Corpus_Uteri
Bladder
Liver
Anorectum
Breast
Thyroid
Pancreas
Testis
Brain
Stomach
Esophagus
Gall_Bladder
AML
Melanoma
Hodgkins
NH_Lymphoma
Vulva.&.Vagina
Penis
ALL
Oropharynx
CML
CLL
Kaposi
Ovary
Lung
Kidney
All_Cancer
Myeloma
Larynx
Cervix
Colorectal
Prostate
Cancer Rate
Percent ..9THC Exposure

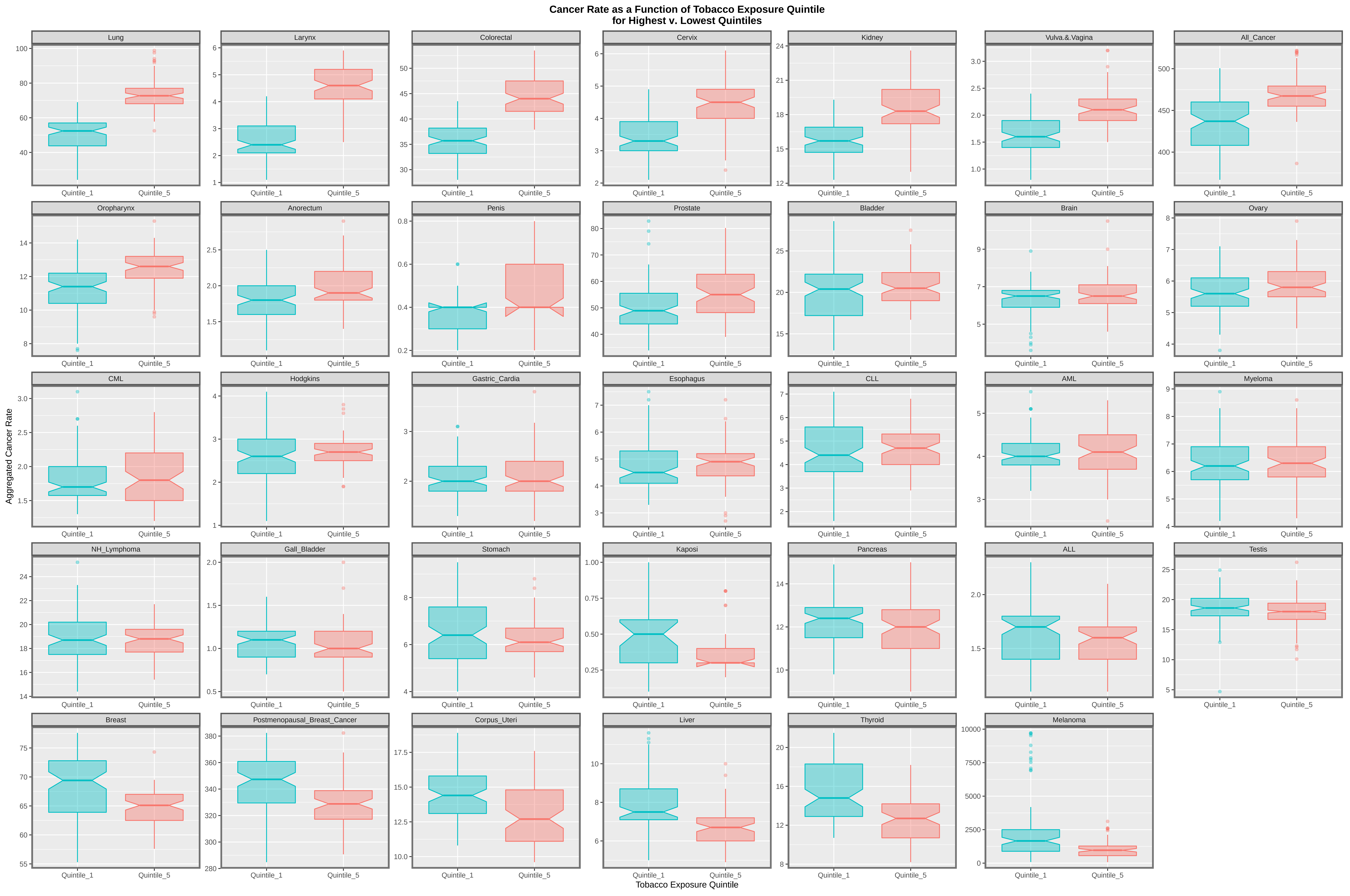
Cancer Rate as a Function of Tobacco Exposure Quintile
for Highest v. Lowest Quintiles
Lung
Larynx
Colorectal
Cervix
Kidney
Vulva.&.Vagina
All_Cancer
Oropharynx
Anorectum
Penis
Prostate
Bladder
Brain
Ovary
CML
Hodgkins
Gastric_Cardia
Esophagus
CLL
AML
Myeloma
NH_Lymphoma
Gall_Bladder
Stomach
Kaposi
Pancreas
ALL
Testis
Breast
Postmenopausal_Breast_Cancer
Corpus_Uteri
Liver
Thyroid
Melanoma
Quintile_1
Quintile_5
Aggregated Cancer Rate
Tobacco Exposure Quintile

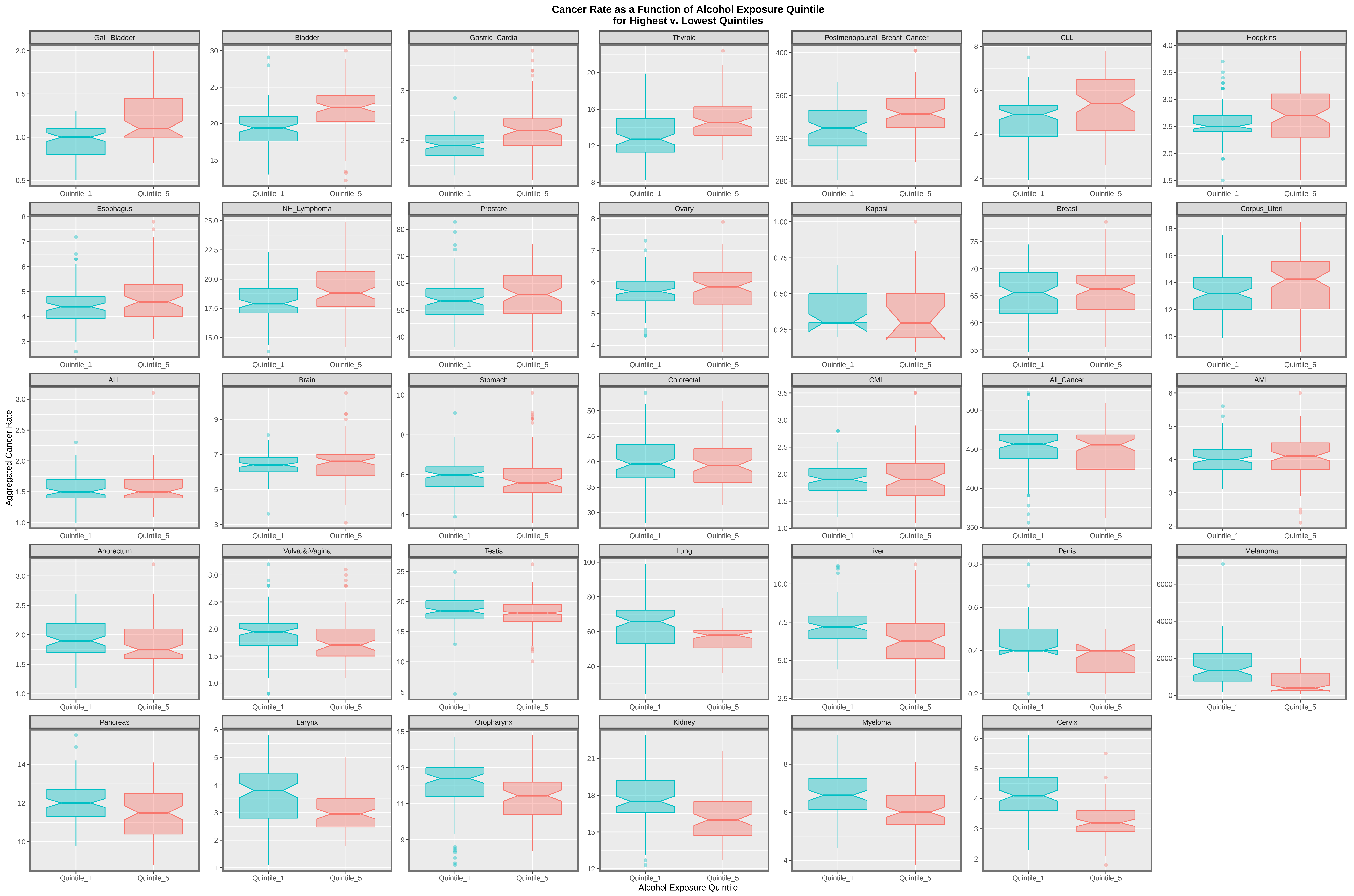
Cancer Rate as a Function of Alcohol Exposure Quintile
for Highest v. Lowest Quintiles
Gall_Bladder
Bladder
Gastric_Cardia
Thyroid
Postmenopausal_Breast_Cancer
CLL
Hodgkins
Esophagus
NH_Lymphoma
Prostate
Ovary
Kaposi
Breast
Corpus_Uteri
ALL
Brain
Stomach
Colorectal
CML
All_Cancer
AML
Anorectum
Vulva.&.Vagina
Testis
Lung
Liver
Penis
Melanoma
Pancreas
Larynx
Oropharynx
Kidney
Myeloma
Cervix
Quintile_1
Quintile_5
Aggregated Cancer Rate
Alcohol Exposure Quintile

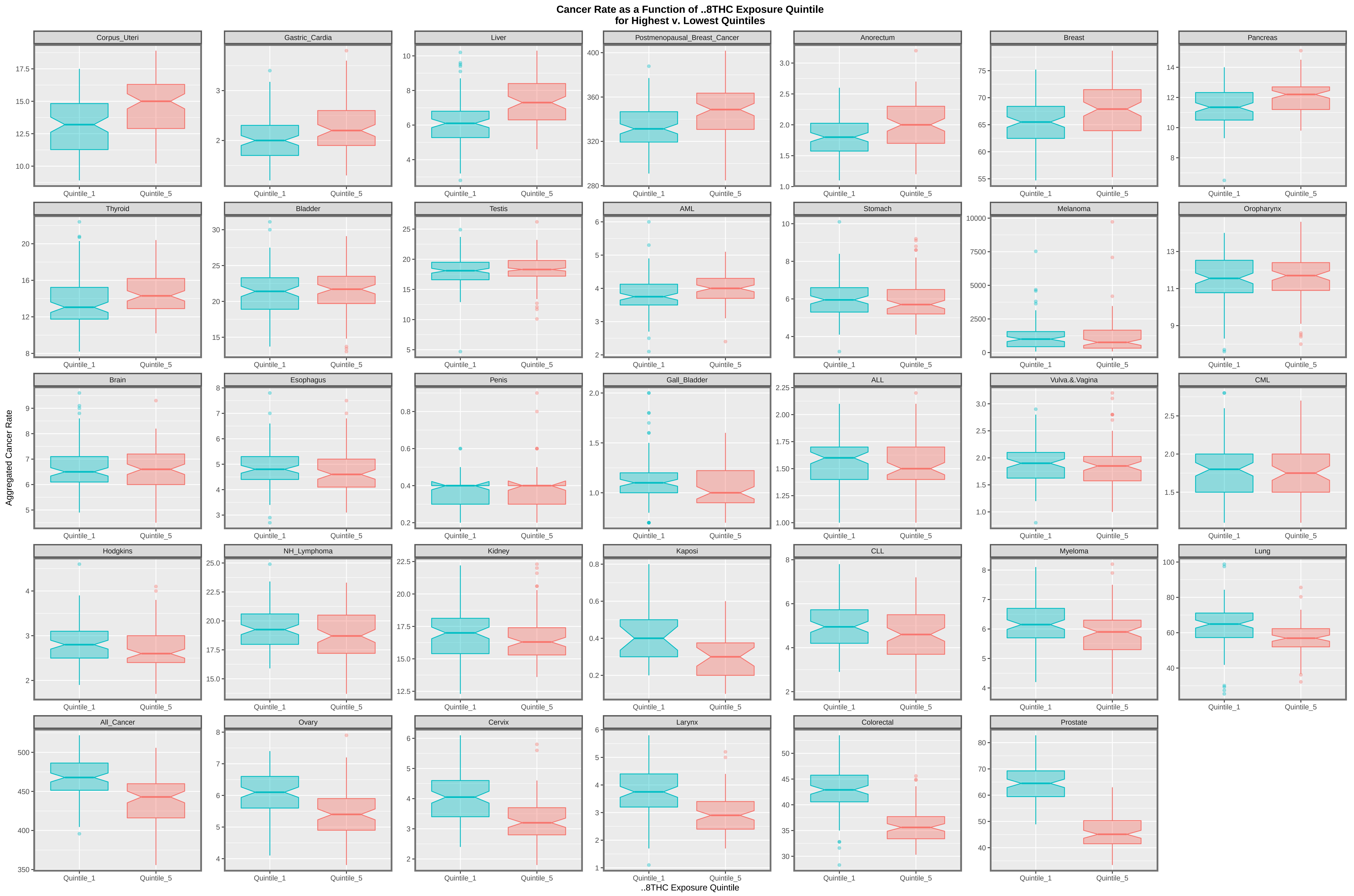
Cancer Rate as a Function of ..8THC Exposure Quintile
for Highest v. Lowest Quintiles
Corpus_Uteri
Gastric_Cardia
Liver
Postmenopausal_Breast_Cancer
Anorectum
Breast
Pancreas
Thyroid
Bladder
Testis
AML
Stomach
Melanoma
Oropharynx
Brain
Esophagus
Penis
Gall_Bladder
ALL
Vulva.&.Vagina
CML
Hodgkins
NH_Lymphoma
Kidney
Kaposi
CLL
Myeloma
Lung
All_Cancer
Ovary
Cervix
Larynx
Colorectal
Prostate
Quintile_1
Quintile_5
Aggregated Cancer Rate
..8THC Exposure Quintile

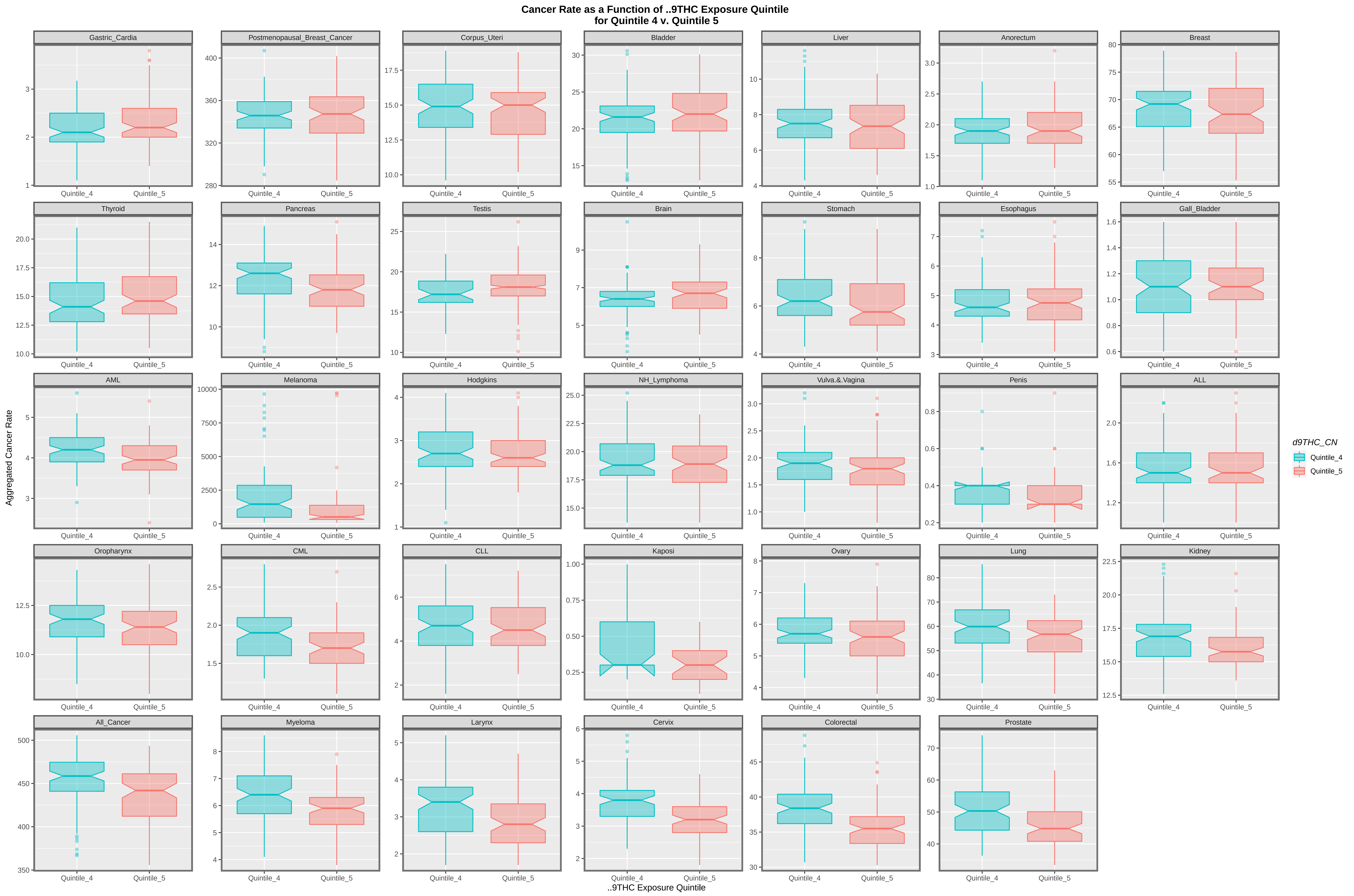
Cancer Rate as a Function of ..9THC Exposure Quintile
for Quintile 4 v. Quintile 5
Gastric_Cardia
Postmenopausal_Breast_Cancer
Corpus_Uteri
Bladder
Liver
Anorectum
Breast
Thyroid
Pancreas
Testis
Brain
Stomach
Esophagus
Gall_Bladder
AML
Melanoma
Hodgkins
NH_Lymphoma
Vulva.&.Vagina
Penis
ALL
Oropharynx
CML
CLL
Kaposi
Ovary
Lung
Kidney
All_Cancer
Myeloma
Larynx
Cervix
Colorectal
Prostate
Quintile_4
Quintile_5
Aggregated Cancer Rate
..9THC Exposure Quintile
d9THC_CN
Quintile_4
Quintile_5

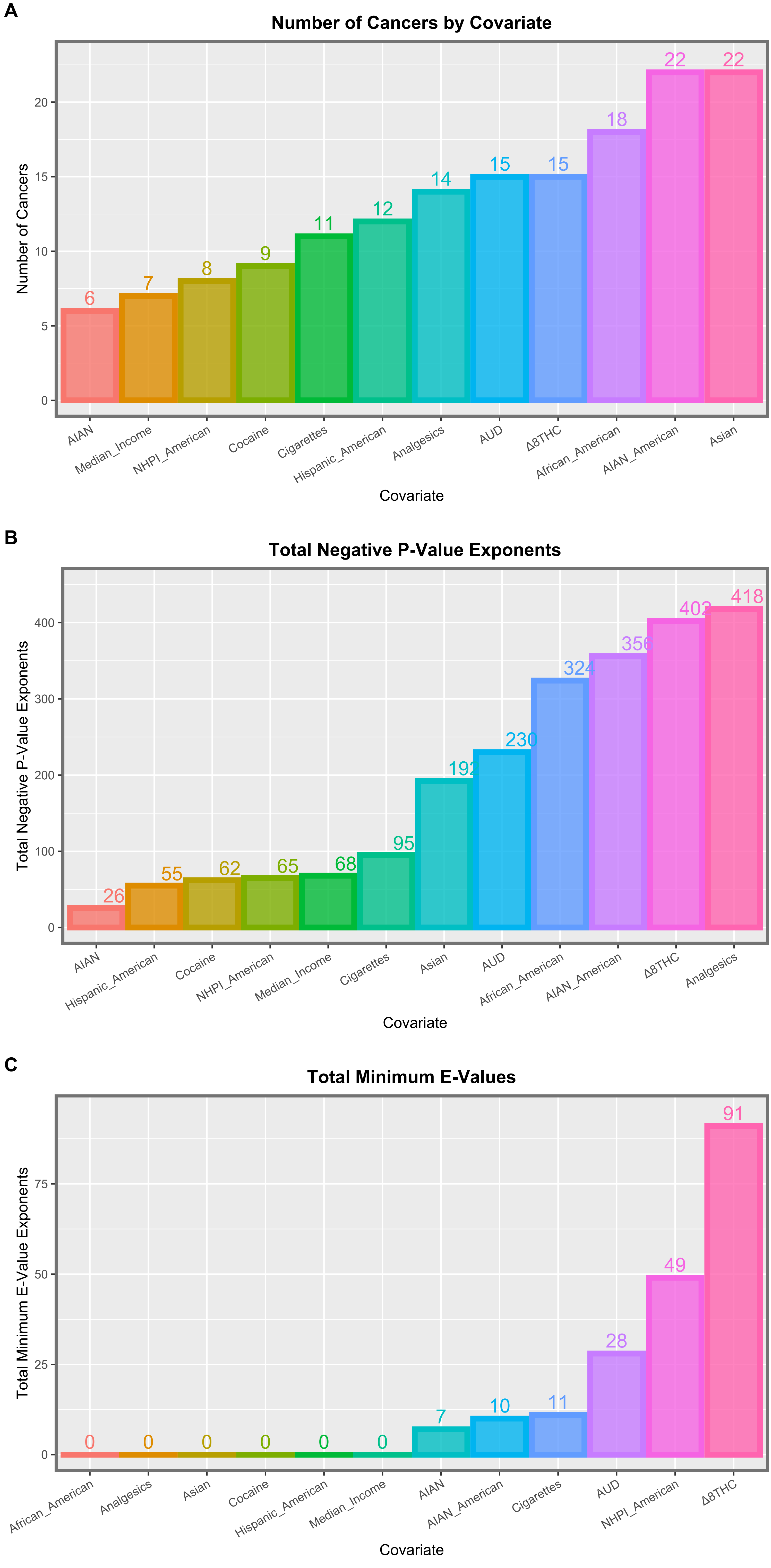
A
Number of Cancers by Covariate
Number of Cancers
Covariate
AIAN
Median_Income
NHPI_American
Cocaine
Cigarettes
Hispanic_American
Analgesics
AUD
Δ8THC
African_American
AIAN_American
Asian
6
7
8
9
11
12
14
15
15
18
22
22
B
Total Negative P-Value Exponents
Total Negative P-Value Exponents
Covariate
AIAN
Hispanic_American
Cocaine
NHPI_American
Median_Income
Cigarettes
Asian
AUD
African_American
AIAN_American
Δ8THC
Analgesics
26
55
62
65
68
95
192
230
324
356
402
418
C
Total Minimum E-Values
Total Minimum E-Value Exponents
Covariate
African_American
Analgesics
Asian
Cocaine
Hispanic_American
Median_Income
AIAN
AIAN_American
Cigarettes
AUD
NHPI_American
Δ8THC
0
0
0
0
0
0
7
10
11
28
49
91

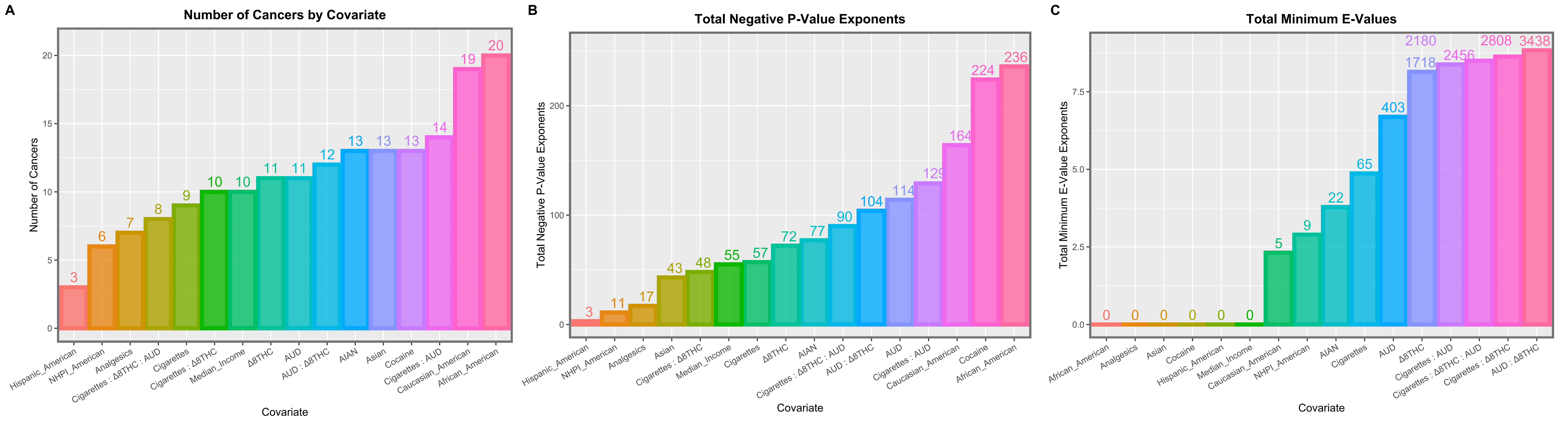
A
Number of Cancers by Covariate
Number of Cancers
Covariate
Hispanic_American
NHPI_American
Analgesics
Cigarettes : Δ8THC : AUD
Cigarettes
Cigarettes : Δ8THC
Median_Income
Δ8THC
AUD
AUD : Δ8THC
AIAN
Asian
Cocaine
Cigarettes : AUD
Caucasian_American
African_American
3
6
7
8
9
10
10
11
11
12
13
13
13
14
19
20
B
Total Negative P-Value Exponents
Total Negative P-Value Exponents
Covariate
Hispanic_American
NHPI_American
Analgesics
Asian
Cigarettes : Δ8THC
Median_Income
Cigarettes
Δ8THC
AIAN
Cigarettes : Δ8THC : AUD
AUD : Δ8THC
AUD
Cigarettes : AUD
Caucasian_American
Cocaine
African_American
3
11
17
43
48
55
57
72
77
90
104
114
129
164
224
236
C
Total Minimum E-Values
Total Minimum E-Value Exponents
Covariate
African_American
Analgesics
Asian
Cocaine
Hispanic_American
Median_Income
Caucasian_American
NHPI_American
AIAN
Cigarettes
AUD
Δ8THC
Cigarettes : AUD
Cigarettes : Δ8THC : AUD
Cigarettes : Δ8THC
AUD : Δ8THC
0
0
0
0
0
0
5
9
22
65
403
1718
2180
2456
2808
3438

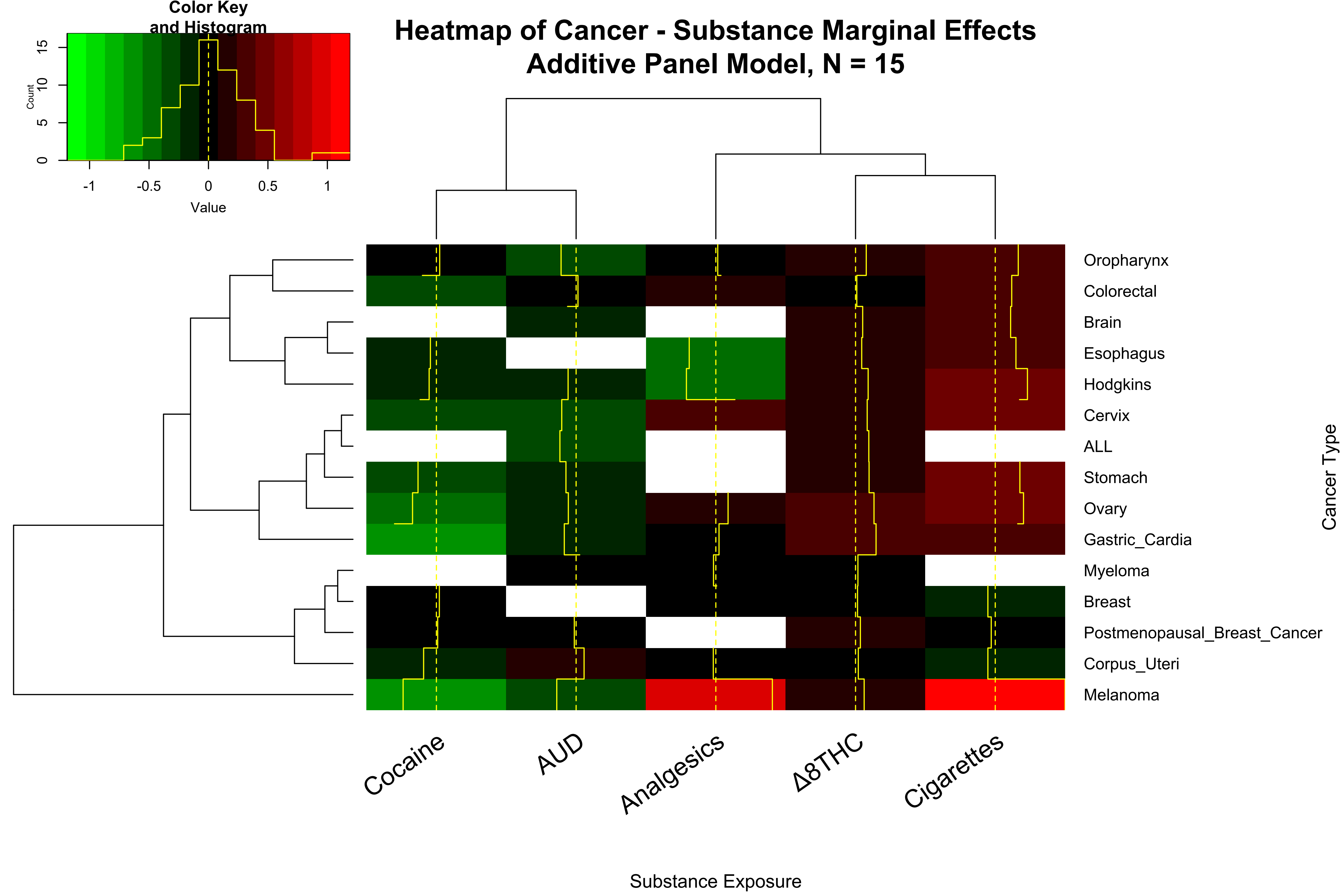
Color Key
and Histogram
Count
0
5
10
15
-1
-0.5
0
0.5
1
Value
Heatmap of Cancer - Substance Marginal Effects
Additive Panel Model, N = 15
Oropharynx
Colorectal
Brain
Esophagus
Hodgkins
Cervix
ALL
Stomach
Ovary
Gastric_Cardia
Myeloma
Breast
Postmenopausal_Breast_Cancer
Corpus_Uteri
Melanoma
Cancer Type
Cocaine
AUD
Analgesics
Δ8THC
Cigarettes
Substance Exposure

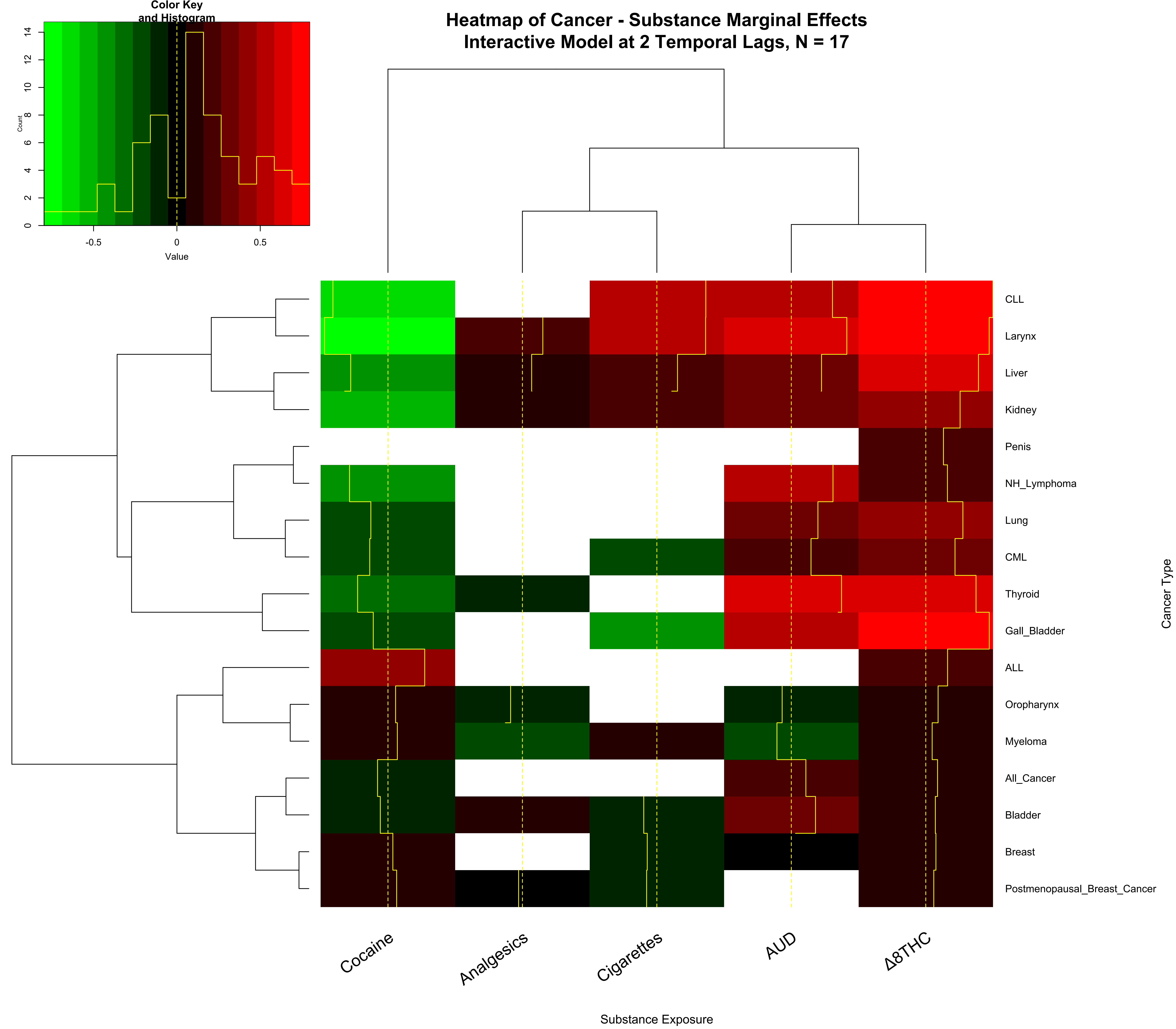
Color Key
and Histogram
Count
Value
-0.5
0
0.5
Heatmap of Cancer - Substance Marginal Effects
Interactive Model at 2 Temporal Lags, N = 17
CLL
Larynx
Liver
Kidney
Penis
NH_Lymphoma
Lung
CML
Thyroid
Gall_Bladder
ALL
Oropharynx
Myeloma
All_Cancer
Bladder
Breast
Postmenopausal_Breast_Cancer
Cancer Type
Cocaine
Analgesics
Cigarettes
AUD
Δ8THC
Substance Exposure

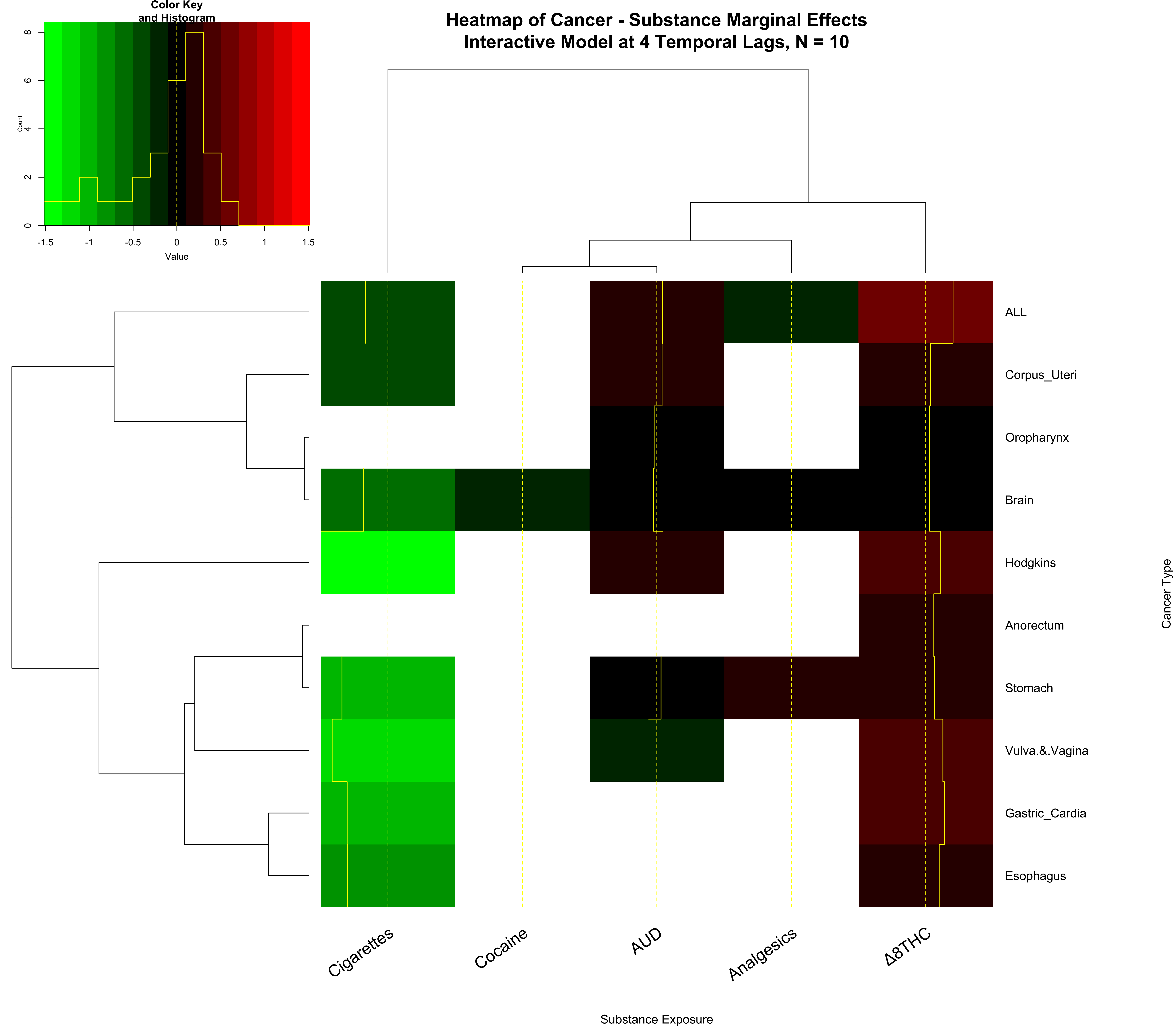
Heatmap of Cancer - Substance Marginal Effects
Interactive Model at 4 Temporal Lags, N = 10
Color Key
and Histogram
Count
Value
-1.5
-1
-0.5
0
0.5
1
1.5
0
2
4
6
8
ALL
Corpus_Uteri
Oropharynx
Brain
Hodgkins
Anorectum
Stomach
Vulva.&.Vagina
Gastric_Cardia
Esophagus
Cancer Type
Cigarettes
Cocaine
AUD
Analgesics
Δ8THC
Substance Exposure
